# N-terminus GTPase domain of the cytoskeleton protein FtsZ plays a critical role in its adaptation to high hydrostatic pressure

**DOI:** 10.3389/fmicb.2024.1441398

**Published:** 2024-08-16

**Authors:** Xue-Hua Cui, Yu-Chen Wei, Xue-Gong Li, Xiao-Qing Qi, Long-Fei Wu, Wei-Jia Zhang

**Affiliations:** ^1^Laboratory of Deep-Sea Microbial Cell Biology, Institute of Deep-Sea Science and Engineering, Chinese Academy of Sciences, Sanya, China; ^2^College of Earth and Planetary Sciences, University of Chinese Academy of Sciences, Beijing, China; ^3^Institution of Deep-Sea Life Sciences, IDSSE-BGI, Sanya, China; ^4^Aix Marseille University, CNRS, LCB, Marseille, France

**Keywords:** obligate piezophile, cell division, cytoskeleton, FtsZ, high hydrostatic pressure, GTPase domain

## Abstract

Studies in model microorganisms showed that cell division is highly vulnerable to high hydrostatic pressure (HHP). Disassembly of FtsZ filaments induced by HHP results in the failure of cell division and formation of filamentous cells in *E. coli*. The specific characteristics of FtsZ that allow for functional cell division in the deep-sea environments, especially in obligate piezophiles that grow exclusively under HHP condition, remain enigmatic. In this study, by using a self-developed HHP *in-situ* fixation apparatus, we investigated the effect of HHP on FtsZ by examining the subcellular localization of GFP-tagged FtsZ *in vivo* and the stability of FtsZ filament *in vitro*. We compared the pressure tolerance of FtsZ proteins from pressure-sensitive strain *Shewanella oneidensis* MR-1 (FtsZ_So_) and obligately piezophilic strain *Shewanella benthica* DB21MT-2 (FtsZ_Sb_). Our findings showed that, unlike FtsZ_So_, HHP hardly affected the Z-ring formation of FtsZ_Sb_, and filaments composed of FtsZ_Sb_ were more stable after incubation under 50 MPa. By constructing chimeric and single amino acid mutated FtsZ proteins, we identified five residues in the N-terminal GTPase domain of FtsZ_Sb_ whose mutation would impair the Z-ring formation under HHP conditions. Overall, these results demonstrate that FtsZ from the obligately piezophilic strain exhibits superior pressure tolerance than its homologue from shallow water species, both *in vivo* and *in vitro*. Differences in pressure tolerance of FtsZ are largely attributed to the N-terminal GTPase domain. This represents the first in-depth study of the adaptation of microbial cytoskeleton protein FtsZ to high hydrostatic pressure, which may provide insights into understanding the complex bioprocess of cell division under extreme environments.

## 1 Introduction

Bacterial division is initiated with Z-ring formation. The cytoskeletal protein FtsZ assembles into a ring-like structure named Z ring at the septum of a dividing cell and serves as a scaffold for the assembly of the divisome (Adams and Errington, 2009). FtsZ is a prokaryotic homologue of tubulin. It consists of an N-terminal tubulin-like core domain that contains GTP binding residues, an intrinsically disordered C-terminal linker region possibly involved in modulating lateral interactions, and a highly conserved short peptide at its C-terminus that interacts with accessory proteins such as FtsA and ZipA, and tethers FtsZ filament to the membrane (Ma and Margolin, 1999; Stricker and Erickson, 2003; Redick et al., 2005; Buske and Levin, 2012, 2013; Gardner et al., 2013; Arjes et al., 2015; Sundararajan and Goley, 2017).

Structural studies suggested that FtsZ are present in open and closed conformations, and switch from closed conformation to open conformation during FtsZ polymerization process. FtsZ polymerizes in a head-to-tail manner, and at the inter-subunit interface forms the catalytic site for GTP hydrolysis. The free FtsZ subunits in closed conformation are prone to assembly into a filament and the hydrolysis of GTP produces GDP-bound FtsZ subunits that are weakened in longitudinal bonds and are prone to disassociate from the other end of the filament (Mukherjee and Lutkenhaus, 1994; Mukherjee, 1998; Redick et al., 2005; Jindal and Panda, 2013; Arjes et al., 2015; Wagstaff et al., 2017). The dynamic treadmilling of FtsZ filaments enables the movement of divisiomes and the even distribution of newly synthesized septal peptidoglycan along the division ring. Mutations of FtsZ with reduced GTPase activity may stabilize the polymer *in vitro* and *in vivo*, and result in a lower rate of treadmilling, an altered septum and peptidoglycan structure, and consequently abnormal cell division (Lu et al., 2001; Stricker et al., 2002; Bisson-Filho et al., 2017; Yang et al., 2017; Monteiro et al., 2018; Perez et al., 2019).

Despite an essential bioprocess, cell division is highly vulnerable to high hydrostatic pressures (HHP). Simulation studies showed that HHP of 30 MPa induced spherical or spindle-shaped epithelial cells, and cell division of *Escherichia coli* was inhibited at 50 MPa and formed filamentous cells (Bourns et al., 1988; Ishii et al., 2004). In these elongated cells, the expression of FtsZ was unaffected, but both Z-ring formation and nucleoids partition were impaired. In addition, the filament of purified FtsZ dissociated *in vitro* as a function of increased pressure. A hypothesis was thus proposed that the failure of FtsZ polymerization and Z-ring formation restrained division of *E.coli* cells under high pressures (Ishii et al., 2004; Kawarai et al., 2004; Aertsen and Michiels, 2005). Similar phenomenon has been observed in eukaryotes. Elevated pressure induced rapid and reversible depolymerization of microtubules in diverse eukaryotic cells (Messier and Seguin, 1978; Arai et al., 2008). *In vitro* studies showed that the application of HHP led to depolymerization of microtubules from both ends, suggesting a decrease of intermolecular interactions between tubulin molecules (Bravim et al., 2010; Hayashi et al., 2016; Takiguchi et al., 2018).

Inhabiting the deep oceanic areas are microbes acclimated to high pressures. They grow preferably (termed as piezophiles) or exclusively (obligate piezophiles) at elevated pressure conditions. To cope with high pressures, deep-sea microbes developed metabolic pathways with higher pressure tolerance, regulation systems responsive to changes in pressure, and cellular membranes with increased proportion of unsaturated fatty acids (Li et al., 1998; Allen et al., 1999; Xiao et al., 2021; Allen and Bartlett, 2022;Tamby et al., 2023). However, the understanding of cell division in deep-sea piezophiles is highly inadequate. Ishii et al. first demonstrated that localization of FtsZ at the septum and the transcription of *ftsZ* in deep-sea strain *Shewanella violacea* DSS12 was not affected by increasing of pressure (Ishii et al., 2002). Kawamoto et al. further showed that despite the presence of Z-rings, deficient of eicosapentaenoic acid (EPA) may lead to defects of cell division and elongated cells under high pressure conditions, suggesting polyunsaturated fatty acid played an important role in the late stage of division under high pressure condition (Kawamoto et al., 2011). Recently we found that HHP impaired the cell division of strain *Tepidibacter hydrothermalis* SWIR-1 isolated from deep-sea hydrothermal vent, while infrared irradiation triggered septum formation and restored cell division through an unknown mechanism (Dai et al., 2024). Collectively, the cellular division under HHP is a complex process affected by many factors, and how is the functional Z-ring formed under high pressure conditions remain unknown. In this study, we analyzed the effect of HHP on FtsZ from pressure sensitive *Shewanella oneidensis* MR-1 (FtsZ_So_) in comparison with FtsZ from hadal obligately piezophilic strain *S. benthica* DB21MT-2 whose optimum growth pressure is 80 MPa (FtsZ_Sb_) (Kato et al., 1998). Comparisons were performed regarding their stability under high pressures *in vivo* and *in vitro*. Furthermore, the amino acids important for the HHP adaptation of FtsZ protein were identified through analyses of chimeric proteins and mutated FtsZ_Sb_ proteins.

## 2 Materials and methods

### 2.1 Strains, plasmids and growth conditions

Strains and plasmids used in this study are listed in Table 1. Strains carrying pDSW plasmids were grown at 37°C in LB medium with ampicillin at 25 μg/mL in liquid cultures and 100 μg/mL in solid cultures. Strains carrying pET plasmids were grown at 37°C in LB medium with 50 μg/mL kanamycin.

**Table 1:** Strains and plasmids used in this study.

Strains and plasmids	Descriptions	Source or reference
**Strains**		
DH5a	F^−^ φ80*lac*ZΔM15 Δ(*lac*ZYA-*arg*F)U169 *rec*A1 *end*A1 *hsd*R17(r_K_^−^, m_K_^+^) *pho*A *sup*E44 λ^−^ *thi*-1 *gyr*A96 *rel*A1	Laboratory collection
EC309	EC251 *ftsZ84*(Ts) *leu*∷Tn10	Michael Tarry et al.
Rosetta™ (DE3)	F-ompT *hsdS*_B_(r_B_^−^ m_B_^−^) *gal dcm* (DE3) pRARE	Laboratory collection
**Plasmids**		
pDSW230	pDSW-FtsZ_Ec_-GFP	David S. Weiss et al.
pDSW2302	pDSW-FtsZ_Sb_-GFP	This study
pDSW2304	pDSW-FtsZ_So_-GFP	This study
pDSW2315	pDSW-FtsZ_So-N_ (FtsZ_So_ 1–316 - FtsZ_Sb_ 317–388)-GFP	This study
pDSW2316	pDSW-FtsZ_So-C_ (FtsZ_Sb_ 1–372 - FtsZ_So_ 380–395)-GFP	This study
pDSW2319	pDSW-FtsZ_So-L_ (FtsZ_Sb_ 1–316 - FtsZ_So_ 317–379 - FtsZ_Sb_ 380–395)-GFP	This study
pDSW2302-1	pDSW-FtsZ_Sb_ S7T-GFP	This study
pDSW2302-2	pDSW-FtsZ_Sb_ T9S-GFP	This study
pDSW2302-3	pDSW-FtsZ_Sb_ S54G-GFP	This study
pDSW2302-4	pDSW-FtsZ_Sb_ T57S-GFP	This study
pDSW2302-5	pDSW-FtsZ_Sb_ I65V-GFP	This study
pDSW2302-6	pDSW-FtsZ_Sb_ I77V-GFP	This study
pDSW2302-7	pDSW-FtsZ_Sb_ L80A-GFP	This study
pDSW2302-8	pDSW-FtsZ_Sb_ D91A-GFP	This study
pDSW2302-9	pDSW-FtsZ_Sb_ E119Q-GFP	This study
pDSW2302-10	pDSW-FtsZ_Sb_ K122R-GFP	This study
pDSW2302-11	pDSW-FtsZ_Sb_ R141K-GFP	This study
pDSW2302-12	pDSW-FtsZ_Sb_ E152A-GFP	This study
pDSW2302-13	pDSW-FtsZ_Sb_ S232R-GFP	This study
pDSW2302-14	pDSW-FtsZ_Sb_ G305D-GFP	This study

### 2.2 Plasmid construction

The DNA fragments of *ftsZ_So_* and *ftsZ_Sb_* without stop codon were amplified from chromosomal DNA of *S. oneidensis* MR-1 and *S. benthica* DB21MT-2, respectively. The *gfp* fragment was amplified from pDSW230 and fused to the 3′ end of *ftsZ_So_* and *ftsZ_Sb_* by over-lap PCR. The generated fragment containing *ftsZ_So_*-*gfp* or *ftsZ_Sb_*-*gfp* were then cloned into the pDSW230 by replacing the *ftsZ_Ec_-gfp* between the XhoI and HindIII sites. Partial fragments of *ftsZ_So_* and *ftsZ_Sb_* were amplified from pDSW2302 and pDSW2304, respectively. By multiple steps of over-lap PCR, the fragments were assembled as follows: *ftsZ_So-N_* contains nucleotides 1–954 from *ftsZ_So_* and nucleotides 955–1,164 from *ftsZ_Sb_*; *ftsZ_So-L_* contains nucleotides 1–954 and 1,120–1,164 from *ftsZ_Sb_* and nucleotides 955–1,140 from *ftsZ_So_*; *ftsZ_So-C_* contains nucleotides 1–1,119 from *ftsZ_Sb_* and nucleotides 1141-1185 from *ftsZ_So_*. The single-amino-acid mutated *ftsZ_Sb_* genes were synthesized by Suzhou Jinweizhi Biotechnology Co., Ltd. The S7T replaces G20 by C, T9S replaces A25 by T, S54G carries GG at position 160 and 161 instead of TC, T57S replaces A169 by T, I65V replaces A193 by G, I77V carries GTG at position 229–231 instead of ATA, L80A carries GC at position 238 and 239 instead of TT, D91A carries CG at position 272 and 273 instead of AT, E119Q replaces G355 by C, K122R replaces A365 by G, R141K replaces G422 by A, E152A replaces A455 by C, S232R replaces A694 by C, and G305D replaces G914 by A. The chimeric and mutated *ftsZ* genes were fused with *gfp* by over-lap PCR and cloned into pDSW230 by replacing the *ftsZ_Ec_-gfp* between the XhoI and HindIII sites. The *ftsZ_So_* and *ftsZ_Sb_* were amplified and cloned into pET28a (+) at BamHI/XhoI sites and generated His-tag at N-terminal.

### 2.3 Western blot analysis

The *E. coli* strains carrying pDSW plasmids were first grown in LB medium at 37°C, atmospheric pressure, for 5 h with shaking. Then the cultures were transferred into sterilized syringes and incubated under atmospheric (0.1 MPa) and high (50 MPa) pressures for 5 h. The syringes were placed in high-pressure vessels (Feiyu Science and Technology Exploitation Co., Ltd., Nantong, China), and the hydrostatic pressure was applied with a water pump (Top Industrie, France). Cells were collected by centrifugation and boiled for 5 min for lysis. The whole cell extracts were separated on 10% SDS-PAGE. The FtsZ-GFP proteins were detected with polyclonal anti-GFP (Thermo Fisher, 1: 10000 dilution), anti-FtsZ_Ec_ (Agisera, 1:3000 dilution) or anti-FtsZ_Sb_ (raised by ChinaPeptides, 1:3000 dilution) as first antibodies, and horseradish peroxidase conjugated goat anti-rabbit IgG as secondary antibody (Thermo Fisher). The CN/DAB substrate kit (Thermo Fisher) was used for the development.

### 2.4 *In-situ* fixation of *E. coli* cells and fluorescent microscope observation

The *E. coli* cells expressing FtsZ-GFP were grown at 37°C for 5 h with shaking. Approximately 500 μL cultures were then transferred into 1 mL syringes containing a small stainless-steel bead. The syringes were assembled into the HHP *in-situ* fixation apparatus and connected with the syringe containing 400 μL 10% paraformaldehyde as fixative. The HHP *in-situ* fixation apparatus were incubated under atmospheric (0.1 MPa) or high (50 MPa) pressures for 5 h before the injection of the fixatives and fixation for 30 min. Ten microliter of fixed cells were taken for microscope observation. The phase-contrast and fluorescence images were taken using BX51 microscope (Olympus). The measurement of cellular length and counting number of cells bearing different types of fluorescence were carried out with Fiji (Schindelin et al., 2012).

### 2.5 Protein purification

The pET plasmids were transformed into Rosetta™ (DE3) cells for protein expression and purification. Cells were grown at 37°C until OD_600nm_ reached 0.4. Isopropyl β-D-thiogalactoside was added to a final concentration of 0.5 mM. After 3 h’ induction, cells were collected by centrifuge and washed twice with 20 mM Tris–HCl pH 8.0. The cell pellet from 1 L culture was resuspended in 100 mL lysis buffer (10 mM imidazole, 20 mM Tris–HCl, 500 mM NaCl) supplemented with Pierce™ protease inhibitor (Thermo Fisher) and lysed through sonication. The cellular lysis was centrifuged at 5000 rpm for 30 min at 4°C, and the supernatant was then loaded to 5 mL Ni^2+^-agarose column (Sangon Biotech) pre-equilibrated with 10 mL lysis buffer. The column was washed with two bed-volumes of washing buffers containing 20 mM and 30 mM imidazole, respectively. The FtsZ was then eluted with 10 mL elution buffer containing 100 mM imidazole. The eluted fractions were analyzed by SDS-PAGE, pooled together, and dialyzed against Tris–HCl pH 8.0 and concentrated using Amicon® Ultra Centrifugal Filters (Millipore). The purified proteins were quantified with Pierce™ BCA Protein Assay Kits (Thermo Fisher).

### 2.6 FtsZ polymerization and TEM observation

*In vitro* polymerization assays were performed as described previously with slight modifications (Ishii et al., 2004). The reactions contained 50 mM KCl, 10 mM MgCl_2_, 50 mM Tris–HCl pH 8.0, 2 mM GTP and 5 μM purified FtsZ proteins. For each assay, 500 μL reaction solution was transferred into 1 mL syringe containing a stainless-steel bead which was assembled into the *in-situ* fixation apparatus with another syringe containing 400 μL 10% glutaraldehyde as fixative. The apparatus was first incubated for 10 min under atmospheric pressure, then pressurized to 50 MPa or remained at 0.1 MPa for 5 h’ incubation. The fixative was injected into the reaction syringe to allow fixation under corresponding pressure conditions for 30 min before decompression. All reactions were performed at ambient temperature. An aliquot of 10 μL fixed polymerization solutions were placed on carbon-coated copper grid and stained with 1% uranyl acetate. At least 8 grids were prepared for each assay and observed by electron microscopy (Hitachi HT7700). The number of FtsZ filaments on each grid were counted.

## 3 Results

### 3.1 Set up of a HHP *in-situ* fixation apparatus

The inhibitory effects of HHP on several bioprocesses, including cell division, are reversible, and the defects restores soon upon decompression (Ishii et al., 2004; Okuno et al., 2013; Hayashi et al., 2016). Therefore, it is important to either carry out the observations under HHP or have the cellular features well-preserved before decompression (Nishiyama et al., 2010; Nishiyama, 2017; Yin et al., 2017; Bourges et al., 2020). *In-situ* HHP fixation systems have been applied by several groups (Chastain and Yayanos, 1991; Ishii et al., 2004; Moussa et al., 2007). In general, the fixative agent and the sample are separated in two chambers by a coverslip or a Parafilm membrane. To achieve effective fixation, the coverslip or membrane need to be broken with a weighted ball or needle and accompanied with rigorous shaking.

**Figure 1:**
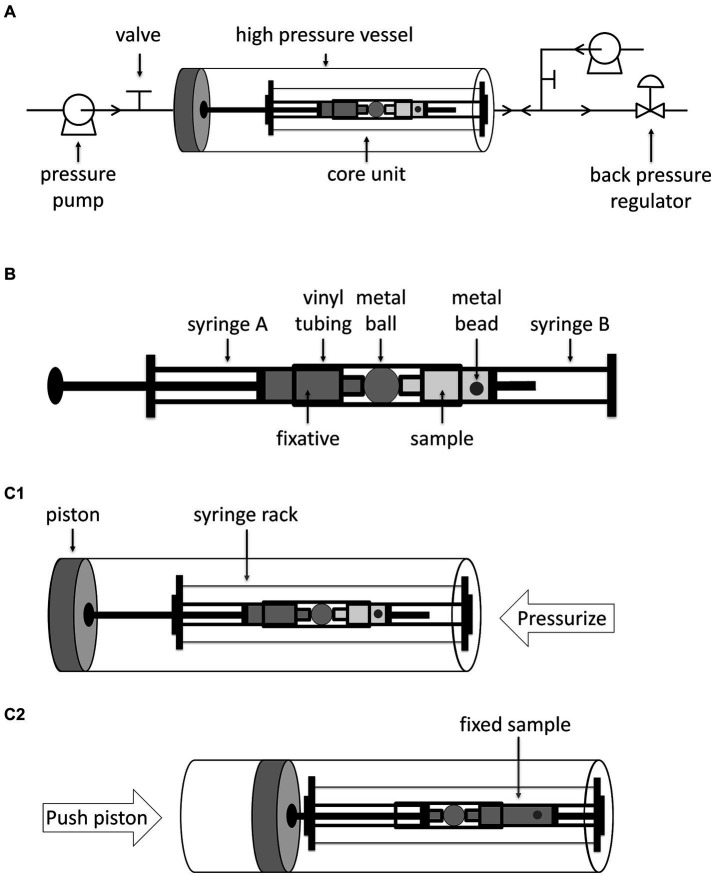
Diagrammatic sketch of *in-situ* fixation apparatus and its operation. Panel **A** shows the *in-situ* fixation apparatus, including the pipelines, the high pressure vessel, the core unit and other accessories. The arrows on the pipelines indicate the flow direction. Panel **B** shows a closer look at the core unit. Panel **C1**,**C2** shows the position of core unit in the high pressure vessel and operation of *in-situ* fixation apparatus. Water is injected from right inlet to pressurize the high pressure vessel. To inject fixative into sample, water is injected from left inlet which pushes the piston as well as the plunger of the syringe A rightwards.

Here, we set up an *in-situ* fixation apparatus optimized for samples with small volumes (Figure 1A). The apparatus consists of a core unit, a pressure vessel, and a pressure controlling system. The core unit constitutes two syringes. Syringe A on the left contains fixative. Syringe B on the right, whose plug is removed, contains the sample to be fixed and a small stainless-steel bead for thorough mixing. The two syringes are separated by a stainless-steel ball, to prevent free exchange of solutions between the two syringes, and the three items are connected by a vinyl tube (Figure 1B and Supplementary Figure S1A). The core unit is installed onto a syringe rack and then placed into the high-pressure vessel (HP vessel) with the plug of syringe A against the piston of the HP vessel. To carry out HHP incubation and fixation, the system is pressurized by injecting water from the inlet on the right using a water pump (Figure 1C1). Upon completion of HHP incubation, water is injected from the inlet on the left, which pushes the piston rightwards, and consequently, injects the fixative from syringe A into syringe B (Figure 1C2). During this process, the pressure inside the HP vessel is maintained by the back pressure regulator. Finally, by inverting the pressure vessel, the fixative and sample in syringe B are thoroughly mixed by the small stainless-steel ball inside. The maximum working pressure of this apparatus is 60 MPa, and three replicates could be manipulated at a time.

To determine the volume of water required to move the piston and the volume of fixative injected into syringe B, we filled syringe A with a colored dye Coomassie blue and syringe B with water, and measured the absorbance of solution in syringe B after HHP incubation and “fixative” injection at 50 MPa (Supplementary Figure S1). By injecting 30 mL or 40 mL water into the HP vessel, the Coomassie blue in syringe A was hardly injected into syringe B (Supplementary Figures S1B,C). When 50 mL or 60 mL water were used, Coomassie blue from syringe A was injected into syringe B, and the final concentration of Coomassie blue in syringe B were 10.2 ± 5.8% and 21.8 ± 3.3%, in relative to the original Coomassie blue solution, respectively (Supplementary Figures S1D,E). Further increase the volume of water injected may risk of bending the syringe rack. Therefore, an injection of 60 mL water was utilized in this study, which guaranteed reproducible fixations with approximately 5 times diluted fixative.

### 3.2 Z-ring formed by FtsZ from piezophilic bacterium has better tolerance to HHP

We first compared the influence of HHP on Z-ring formation of three FtsZ proteins from strains with different pressure tolerance: one from an obligate piezophile that grown exclusively at HHP condition (FtsZ_Sb_ from *S. benthica* DB21MT-2) and two from pressure sensitive strains (FtsZ_Ec_ from *E. coli*, and FtsZ_So_ from *S. oneidensis* MR-1). The FtsZ proteins were expressed in *E. coli* with a GFP-tag at C-terminal. The *E. coli* cells were grown aerobically under atmospheric pressure to allow maturation of fluorescent GFP before incubated under 0.1 MPa or 50 MPa, respectively. The expressions of FtsZ-GFP fusion proteins were confirmed by western-blot analysis. All three fusion proteins (FtsZ_Ec_-GFP, FtsZ_So_-GFP and FtsZ_Sb_-GFP) were expressed at comparable level and no free GFP was detected suggesting that the fusion proteins remained stable under both pressure conditions, and the green fluorescence is exclusively related to the heterogeneously expressed FtsZs (Supplementary Figure S2A).

**Figure 2:**
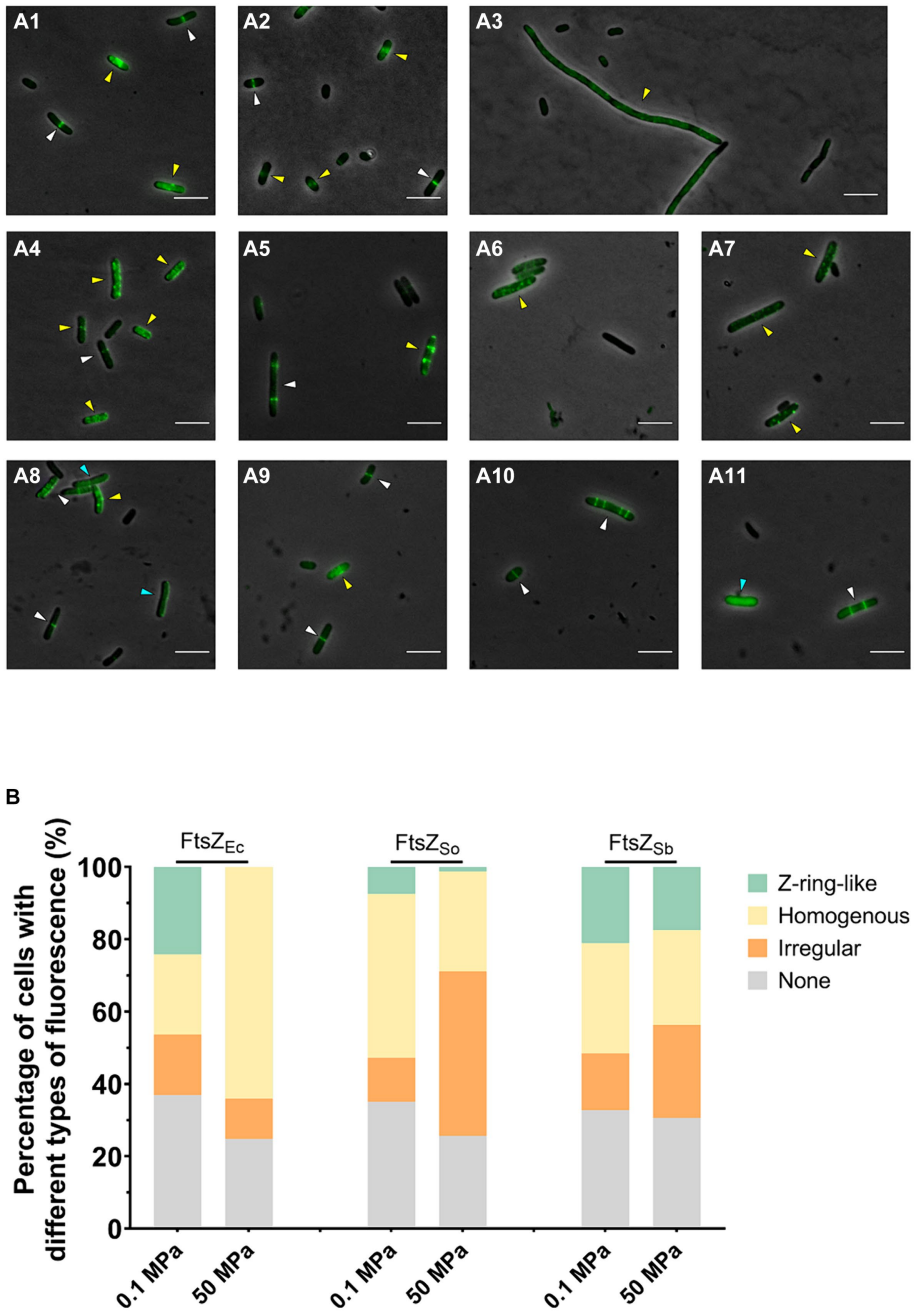
Effect of HHP on subcellular localization of FtsZ_Ec_, FtsZ_So_ and FtsZ_Sb_. Panel **A** shows fluorescence in *E. coli* cells expressing different FtsZ proteins under ambient and high pressures. Panel **A1–A3** show cells expressing FtsZ_Ec_-GFP incubated under 0.1 MPa **(A1,A2)** and 50 MPa **(A3)**; Panel **A4–A7** show cells expressing FtsZ_So_-GFP incubated under 0.1 MPa **(A4,A5)** and 50 MPa **(A6,A7)**; Panel **A8–A11** show cells expressing FtsZ_Sb_-GFP incubated under 0.1 MPa **(A8,A9)** and 50 MPa **(A10,A11)**. All scale bars indicate 5 μm. The white arrows indicate cells with Z-ring-like fluorescence, blue arrows indicate cells with homogenous fluorescence and yellow arrows indicate cells with ectopic fluorescence. Panel **B** shows the average percentage of cells with different types of fluorescence. The data were collected from three independent assays. In each assay, over 300 cells were analyzed for each culture.

After 5 h’ incubation under atmospheric pressure, the *E. coli* cells expressing all three FtsZ-GFP fusion proteins were mostly 2–4 μm short-rod shaped. Consistent with previous observation, incubation at 50 MPa impeded division of cells expressing FtsZ_Ec_-GFP, and the majority of cells were filamentous with an average cellular length over 20 μm. In contrast, cells expressing FtsZ_So_-GFP and FtsZ_Sb_-GFP remained short-rod shaped at incubation at 50 MPa, and the majority of cells were 2–6 μm in length (Figure 2 and Supplementary Figure S3).

The subcellular localization of different FtsZ proteins were examined with fluorescent microscope. In all three cultures tested, over 60% cells were fluorescent, and three types of fluorescence were observed. The sharp bands perpendicular to the long axis of cell was referred to as Z-ring-like fluorescence, which were most likely properly polymerized and located FtsZ-GFP filaments (Figures 2A1–A11, white arrows). The sporadic spots, spiral and filamentous patterns were collectively recognized as ectopic fluorescence, which indicated abnormal polymerization or localization of FtsZ-GFP (Figures 2A1–A11, yellow arrows). The homogenous fluorescence indicated free FtsZ-GFP subunits failed to polymerize (Figures 2A1–A11, blue arrows). When FtsZ_Ec_-GFP was expressed, cells with Z-ring-like or homogenous fluorescence each accounted for 20% in cultures incubated under atmospheric pressure, while cells with Z-ring-like fluorescence can hardly be observed after incubation under 50 MPa and the percentage of cells with homogenous fluorescence increased remarkably to approximately 60% (Figures 2A1–A1,B). In cells expressing FtsZ_So_-GFP, the proportion of cells with Z-ring-like fluorescence decreased from 7% under atmospheric pressure to around 1% under 50 MPa, along with an increase of cells with ectopic fluorescence from 12 to 46% (Figures 2A4–A7,B). On the contrary, the fluorescence patterns of cells expressing FtsZ_Sb_-GFP were stable under both pressures. Cells exhibiting Z-ring-like fluorescence accounted for 21.1% at 0.1 MPa and 17.5% at 50 MPa, respectively (Figures 2A8–A11,B). These observations suggested that both FtsZ_So_ and FtsZ_Sb_ were able to form or incorporate into Z-ring under atmospheric pressure. Moreover, unlike the Z-rings composed of FtsZ from pressure-sensitive strains, which were prone to depolymerize or aggregate under high pressure conditions, the Z-rings composed of FtsZ from the obligate piezophile (FtsZ_Sb_) were more resistant to high pressure *in vivo*.

### 3.3 FtsZ_Sb_ filaments are more stable under high pressures *in vitro*

**Figure 3:**
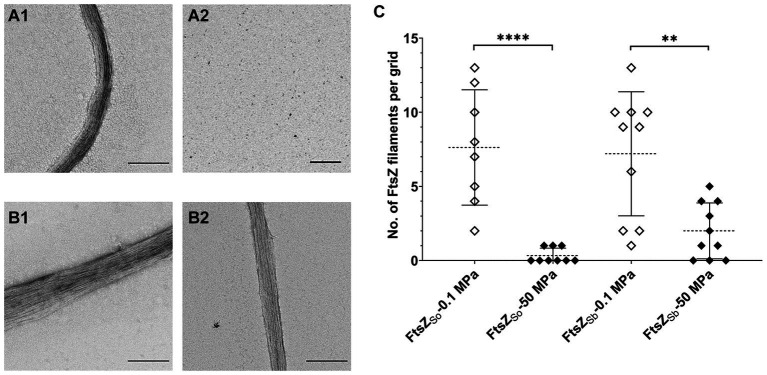
Effect of HHP on FtsZ polymerization *in vitro*. Panel **A** shows filaments of purified FtsZ_So_ incubated at 0.1 MPa **(A1)** and 50 MPa **(A2)**, panel **B** shows filaments of purified FtsZ_Sb_ incubated at 0.1 MPa **(B1)** and 50 MPa **(B2)**, respectively. The scale bar indicates 2 μm. Panel **C** shows number of FtsZ filaments observed on each grid. Mean value and standard deviation are indicated by dashed lines and solid lines. Statistical analysis was carried out using two-tailed unpaired t-test: **** *p* < 0.0001, ** *p* < 0.01.

Both the intrinsic property of FtsZ itself and its interaction with division factors such as ZapAB affect stability of FtsZ polymer *in vivo* (Ebersbach et al., 2008; Durand-Heredia et al., 2011; Galli and Gerdes, 2012). Here we focused on the influence of HHP on FtsZ polymerization and with purified FtsZ proteins we examined the stability of different FtsZ filaments *in vitro*. Upon incubation under atmospheric pressure condition, bundles of filaments could be observed for both FtsZ_So_ and FtsZ_Sb_. To be more specific, the average numbers of filaments per grid were 7.6 and 7.2, for FtsZ_So_ and FtsZ_Sb_, respectively (Figures 3A1,B1,C). After incubation at 50 MPa, filament of FtsZ_So_ was hardly observed, we found only 3 bundles of FtsZ_So_ filaments from 9 grids, while 20 bundles of FtsZ_Sb_ filaments were found on 10 grids (Figures 3A2,B2,C). Although the number of filament bundles cannot be used to quantify polymerization of FtsZ subunits, the apparent different performance between FtsZ_So_ and FtsZ_Sb_ filaments suggested a higher stability of FtsZ_Sb_ filaments or stronger polymerization of FtsZ_Sb_ under HHP conditions. Moreover, the difference between FtsZ_So_ and FtsZ_Sb_ was determined by their intrinsic properties.

### 3.4 N-terminal GTPase domain plays an important role in HHP adaptation of FtsZ_Sb_

**Figure 4:**
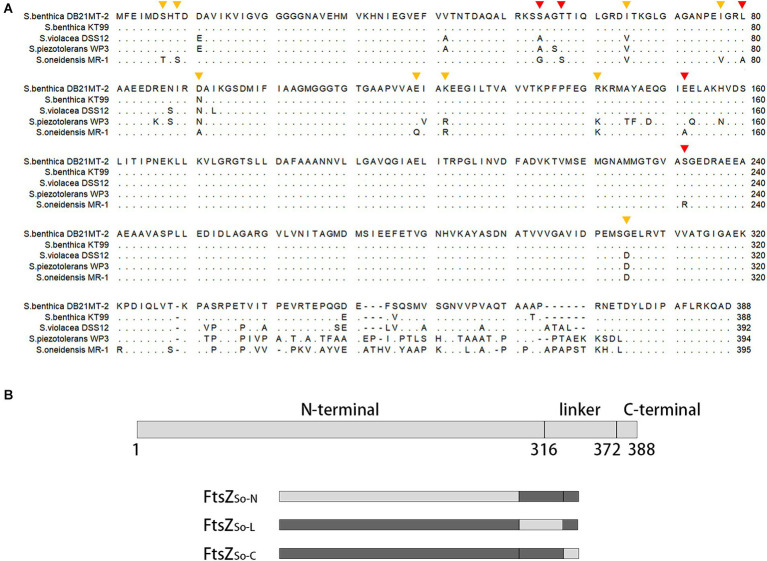
Alignment of FtsZ proteins from *Shewanella* strains with different pressure tolerance and diagram of chimeric FtsZ construction. Panel **A** shows alignment of FtsZ proteins from obligate piezophilic strains *S. benthica* DB21MT-2 and KT99, piezophilic strain *S. violacea* DSS12, pressure tolerant strain *S. piezotolerans* WP3 and pressure sensitive strain *S. oneidensis* MR-1. Identical amino acids are present as dots. The mutated amino acids are marked with triangles, the orange ones indicate mutations with similar proportion of Z-ring-like fluorescence under different pressures, and the red ones indicate mutants with less Z-ring-like fluorescence under high pressures. Panel **B** shows three conserved regions in FtsZ and composition of chimeric FtsZ proteins. The number beneath indicate amino acid position based on the sequence of FtsZ_Sb_. Boxes in light grey indicate fragments cloned from pressure sensitive FtsZ_So_ and boxes in dark grey indicate those cloned from pressure tolerant FtsZ_Sb_.

Both *in vivo* and *in vitro* assays suggested that FtsZ from obligate piezophile (FtsZ_Sb_) was better adapted to HHP than its counterpart from pressure sensitive strain (FtsZ_So_). A sequence alignment was performed using FtsZ proteins from 5 representative *Shewanella* strains with different pressure tolerance: the obligate piezophilic strains DB21MT-2 and KT99, piezophilic strain DSS12, pressure tolerant strain WP3 and pressure sensitive strain MR-1 (Figure 4A). They all comprised three domains: a highly conserved N-terminal GTPase domain (amino acids 1 to 316, the numbers are based on the sequence of FtsZ_Sb_), a divergent linker region (amino acids 317 to 372), and a conserved C-terminal domain (amino acids 373 to 388). To understand the contribution of each domain to HHP adaptation, we constructed three chimeric FtsZ proteins by replacing each of the three domains in pressure-tolerant FtsZ_Sb_ with corresponding segment from pressure sensitive FtsZ_So_, and resulted in FtsZ_So-N_, FtsZ_So-L_ and FtsZ_So-C_, respectively (Table 1 and Figure 4B).

The three chimeric FtsZ proteins were expressed with GFP tag in *E. coli* cells (Supplementary Figure S2B). Similar to cells expressing the wild-type FtsZ_Sb_, cells containing chimeric FtsZ proteins were short-rod shaped, and the majority (around 80%) were fluorescent (Supplementary Figure S4). Under atmospheric pressure, cells expressing FtsZ_So-N_-GFP and FtsZ_So-L_-GFP had similar fluorescent patterns: cells exhibiting Z-ring-like, ectopic and homogenous fluorescence accounted for 10, 30 and 40%, respectively. In cells expressing FtsZ_So-C_-GFP, the proportion of Z-ring-like and ectopic fluorescent cells were relatively lower (accounted for 5 and 16%, respectively) while homogenous fluorescent cells increased to approximately 60% (Table 2). After incubation under 50 MPa, Z-ring-like fluorescent cells could be observed in all three strains, but their proportions were affected differently by HHP (Table 2). In cells expressing FtsZ_So-N_-GFP, incubation at 50 MPa clearly decreased the ratio of Z-ring-like fluorescent cells (10.2 ± 4.5% at 0.1 MPa versus 5.8 ± 1.4% at 50 MPa), along with an increase of the cells with ectopic fluorescence (33.1 ± 13.3% at 0.1 MPa versus 43.9 ± 4.6% at 50 MPa). When FtsZ_So-L_-GFP was expressed, the fluorescent profile was hardly affected by changing of pressure, which was similar to the wild-type FtsZ_Sb_-GFP. Meanwhile, a higher ratio of cells with Z-ring-like fluorescence was observed under HHP condition when FtsZ_So-C_-GFP was expressed (4.7 ± 1.1% at 0.1 MPa versus 9.6 ± 3.5% at 50 MPa). These observations suggested that, compared to FtsZ_So_, the higher HHP tolerance of Z-ring formed by FtsZ_Sb_ was largely due to its N-terminal GTPase domain. The linker domain hardly affected the HHP tolerance, while the C-terminal domain from FtsZ_So_ may increase the HHP stability of Z-ring *in vivo*.

**Table 2:** Sub-cellular localization of chimeric FtsZ under different pressure conditions.

FtsZ	Pressures (MPa)	Ratio of different types of fluorescence (%)				No. of cells analyzed
		Z-ring-like	Homogenous	Ectopic	None	
FtsZ_So-N_	0.1	10.2 ± 4.5	37.0 ± 16.9	33.1 ± 13.3	19.7 ± 8.4	985
	50	5.8 ± 1.4	34.5 ± 6.4	43.9 ± 4.6	15.8 ± 9.7	1,043
FtsZ_So-L_	0.1	11.9 ± 6.3	36.5 ± 4.5	27.8 ± 5.9	23.7 ± 14.8	1,038
	50	11.3 ± 4.1	37.7 ± 3.6	26.0 ± 6.0	25.0 ± 11.2	1,021
FtsZ_So-C_	0.1	4.7 ± 1.1	59.8 ± 3.6	15.6 ± 9.7	19.8 ± 12.1	698
	50	9.6 ± 3.5	54.2 ± 0.6	14.6 ± 7.5	21.6 ± 11.6	790

**Figure 5:**
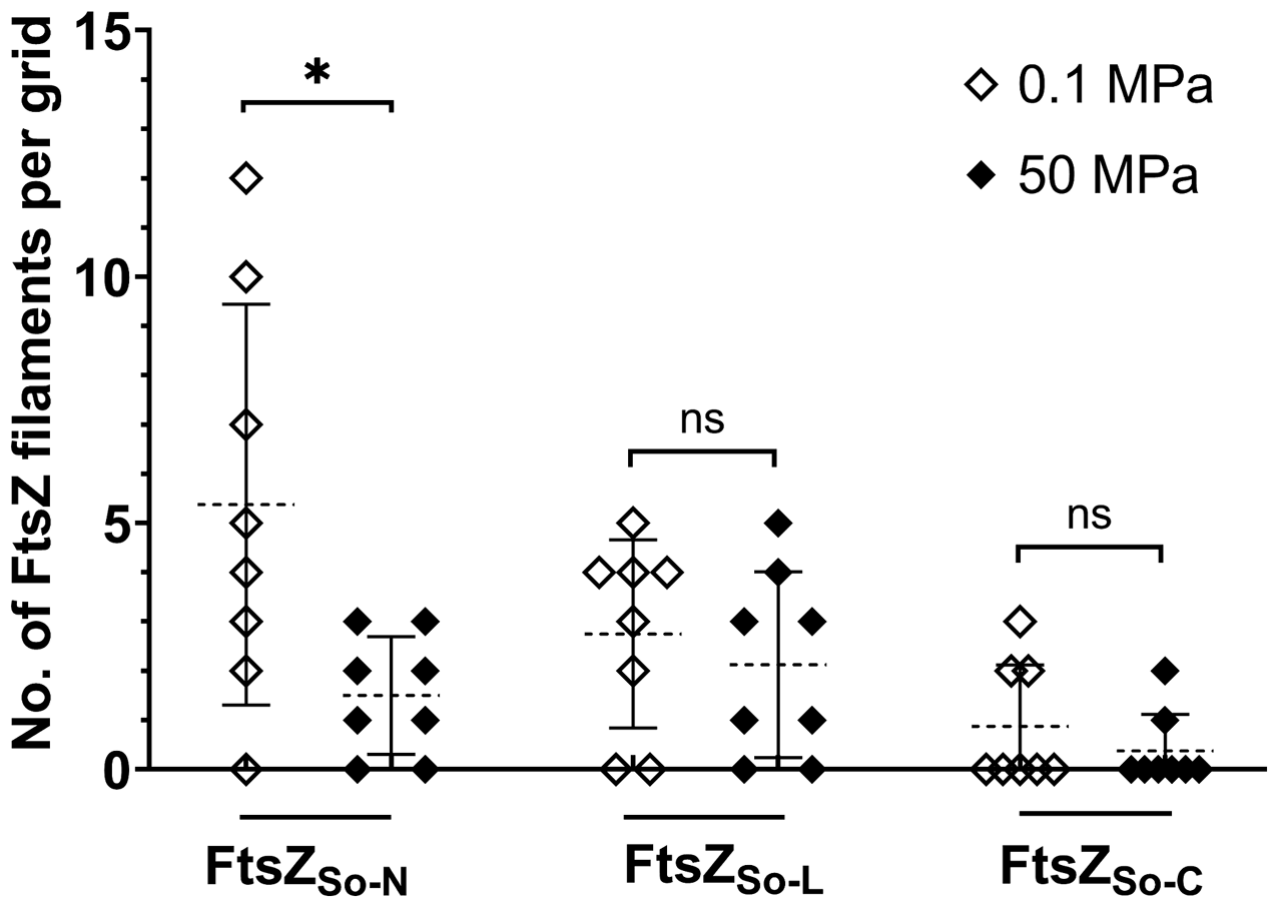
Effect of HHP on chimeric FtsZ polymerization *in vitro.* Mean value and standard deviation are indicated by dashed lines and solid lines. Statistical analysis was carried out using two-tailed unpaired t-test: * *p* < 0.05, ns *p* > 0.05.

The HHP stability of chimeric FtsZ filaments was further examined *in vitro*. For samples incubated under atmospheric pressure, filament bundles of FtsZ_So-N_ and FtsZ_So-L_ could be observed on most grids, while filaments of FtsZ_So-C_ can only be found occasionally. As expected, the influence of elevated pressure varied from different chimeric FtsZ proteins (Figure 5). At atmospheric pressure, an average of 5.4 bundles of filaments composed of FtsZ_So-N_ could be found on a grid, and in one case, a maximum of 12 bundles of filaments were observed. Meanwhile, no more than three bundles of filaments could be visualized with FtsZ_So-N_ incubated under 50 MPa. For filaments formed by FtsZ_So-L_ and FtsZ_So-C_, no significant difference was found between incubation at atmospheric and high pressure conditions. Collectively, these results indicated that the N-terminal GTPase domain played an important role in Z-ring formation and polymerization of FtsZ filaments under high pressures.

### 3.5 Mutation of five amino acids from N-terminal GTPase domain reduces pressure tolerance of FtsZ_Sb_

**Figure 6:**
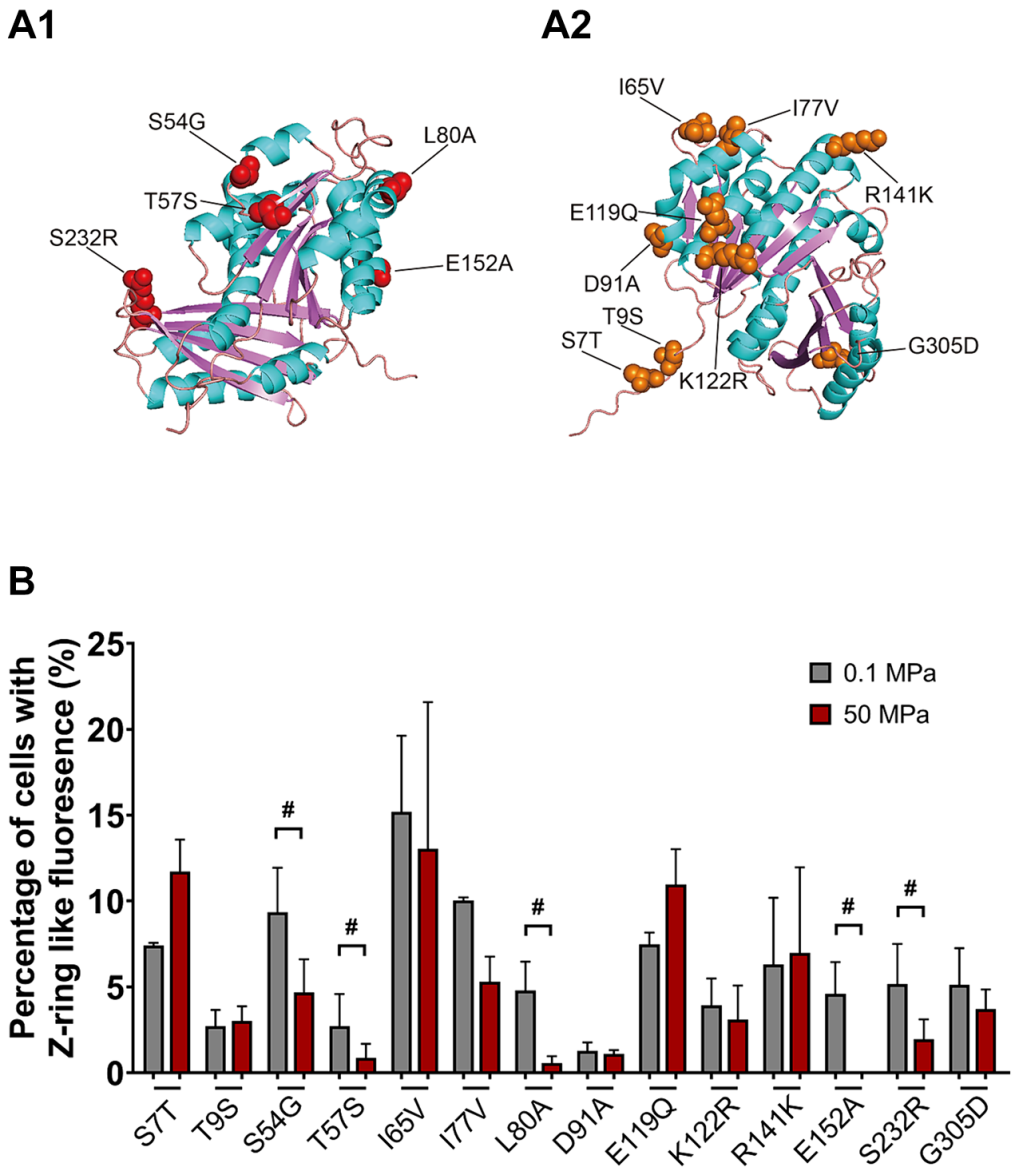
Mapping of mutants on the predicted structure of FtsZ_Sb_ and the effect of HHP on subcellular localization of mutated FtsZ_Sb_. Panel **A1,A2** show the mutated amino acids on FtsZ_Sb_. The amino acid numbers refer to the FtsZ_Sb_ sequence. Panel **B** shows the ratio of cells exhibiting Z-ring-like fluorescence under 0.1 MPa (grey bars) and under 50 MPa (red bars). The mutated FtsZ_Sb_ whose localization was severely affected by high pressure were marked with #.

A closer examination of the N-terminal GTPase domains from FtsZ_So_ and FtsZ_Sb_ showed 14 different amino acids, all of them were exposed on the surface (indicated by orange and red arrows in Figure 4A and Figures 6A1,A2). By site-directed mutagenesis, we replaced each of the 14 amino acids in FtsZ_Sb_ with the corresponding amino acid from FtsZ_So_ (Table 1). Western-blot showed that the expression and stability of mutant FtsZ_Sb_-GFP fusion proteins in *E. coli* cells were not affected by elevated pressure (Supplementary Figure S2C). The localization of the mutated FtsZ_Sb_ at 0.1 MPa and 50 MPa were then examined (Figure 6B and Table 3). In general, cells with Z-ring-like fluorescence accounted for 5 to 15% under atmospheric pressure condition, which was lower compared to cells expressing the wild-type FtsZ_Sb_ protein (approximately 20%). Among all the mutant FtsZ_Sb_, D91A had the lowest ratio of Z-ring-like fluorescent cells of 1.3% under atmospheric pressure, and higher amount of non-fluorescent cell and cells with ectopic fluorescence. Mutant T9S and T57S were also deficient in Z-ring formation under atmospheric pressure, the ratio of Z-ring-like fluorescent cell was 2.7%, and over half of the populations exhibited homogenous fluorescence.

Upon incubation under HHP, the ratio of cells with Z-ring-like fluorescent slightly increased in mutant S7T and E119Q, from around 7 to 11%. For the rest of the mutants, the ratio of cells exhibiting Z-ring-like fluorescence decreased at different degrees. In 7 mutants (T9S, I65V, I77V, D91A, K122R, R141K, and G305D), cultures at 50 MPa and 0.1 MPa had comparable ratio (less than 2-fold changes) of Z-ring-like fluorescent cell. It suggested that elevated pressure had little influence on their Z-ring formation, resembling the wild-type FtsZ_Sb_ protein. On the other hand, HHP decreased the ratio of cells exhibiting Z-ring-like fluorescence by over 2-fold in the other five mutants (S54G, T57S, L80A, E152A, and S232R), indicating a more critical role of these amino acids in maintaining the Z-ring structure under HHP condition. Remarkably, in cultures of L80A and E152A, around 5% cells bore Z-ring-like fluorescence under atmospheric pressure, while the ratio decreased to less than 1% under high pressure condition.

**Table 3:** Effect of HHP on sub-cellular localization of mutated FtsZ_Sb_.

FtsZ_Sb_ mutant	Pressures (MPa)	Ratio of different types of fluorescence (%)				No. of cells analyzed
		Septum	Homogenous	Ectopic	None	
S7T	0.1	7.4 ± 0.1	40.1 ± 3.3	41.1 ± 9.6	11.4 ± 6.1	687
	50	11.7 ± 1.8	43.3 ± 14.7	31.0 ± 18.4	14.0 ± 5.6	713
T9S	0.1	2.7 ± 0.9	59.3 ± 0.8	25.1 ± 9.3	12.9 ± 9.5	695
	50	3.0 ± 0.8	57.4 ± 2.4	20.6 ± 6.4	19.0 ± 4.8	689
S54G^#^	0.1	9.4 ± 2.6	60.0 ± 5.1	15.3 ± 3.6	15.3 ± 5.5	996
	50	4.7 ± 1.9	65.4 ± 9.7	20.9 ± 5.2	9.0 ± 6.5	920
T57S^#^	0.1	2.7 ± 1.9	51.8 ± 11.3	22.1 ± 11.4	23.4 ± 17.5	1,026
	50	0.9 ± 0.8	71.2 ± 7.6	14.0 ± 6.0	13.9 ± 3.9	1,056
I65V	0.1	15.2 ± 4.4	40.8 ± 2.8	25.0 ± 21.3	19.0 ± 14.0	618
	50	13.0 ± 8.5	56.4 ± 9.5	26.0 ± 18.2	4.5 ± 0.1	618
I77V	0.1	10.0 ± 0.2	49.4 ± 20.9	23.7 ± 12.7	16.9 ± 8.0	628
	50	5.3 ± 1.5	61.9 ± 25.4	19.3 ± 13.5	13.4 ± 10.4	660
L80A^#^	0.1	4.8 ± 1.7	40.4 ± 19.3	16.6 ± 3.9	38.2 ± 24.9	730
	50	0.6 ± 0.4	57.0 ± 10.0	18.2 ± 5.5	24.4 ± 4.0	674
D91A	0.1	1.3 ± 0.5	26.6 ± 8.7	33.5 ± 6.0	38.7 ± 2.2	626
	50	1.1 ± 0.2	37.9 ± 4.7	19.8 ± 4.6	41.2 ± 0.1	641
E119Q	0.1	7.5 ± 0.7	51.5 ± 1.9	24.8 ± 1.4	16.2 ± 4.0	629
	50	11.0 ± 2.0	45.0 ± 3.7	13.6 ± 0.5	30.4 ± 6.2	729
K122R	0.1	5.3 ± 2.6	36.6 ± 13.0	21.5 ± 8.6	36.7 ± 8.2	674
	50	3.5 ± 1.6	52.6 ± 15.5	20.9 ± 6.6	23.1 ± 13.1	663
R141K	0.1	6.3 ± 3.9	39.9 ± 11.1	22.7 ± 1.4	31.1 ± 8.6	617
	50	7.0 ± 5.0	46.3 ± 7.9	13.3 ± 0.6	33.4 ± 2.3	632
E152A^#^	0.1	4.6 ± 1.9	46.1 ± 22.1	23.4 ± 7.7	25.8 ± 12.6	671
	50	0.0 ± 0.0	58.5 ± 21.0	13.2 ± 1.6	28.3 ± 22.6	666
S232R^#^	0.1	6.5 ± 0.6	69.5 ± 6.0	9.4 ± 4.0	14.6 ± 2.6	949
	50	2.4 ± 1.1	75.4 ± 2.4	12.5 ± 3.3	9.6 ± 6.8	924
G305D	0.1	5.1 ± 2.1	50.2 ± 17.6	19.7 ± 1.0	25.0 ± 18.7	636
	50	3.7 ± 1.1	46.8 ± 2.6	13.6 ± 3.2	35.9 ± 6.9	619

^#^Indicated mutated FtsZ_Sb_ that form less Z-ring-like fluorescence under HHP condition.

Collectively, by applying a novel HHP *in-situ* fixation apparatus, we examined the pressure tolerance of cytoskeleton protein FtsZ from bacterial strains inhabiting different water depth. Our results suggested that FtsZ_Sb_ from obligately piezophilic strain *S. benthica* DB21MT-2 inhabiting the bottom of the Mariana Trench has better pressure tolerance than its homologues from shallow water species with respect to Z-ring formation *in vivo* and FtsZ polymerization *in vitro*. Differences in pressure tolerance of FtsZ was largely attributed to the N-terminal GTPase domain and five amino acids were probably involved in the adaptation of FtsZ to the HHP environment.

## 4 Discussion

It is now established that the cytoskeleton of both prokaryotes and eukaryotes are vulnerable to HHP, with microtubules dissociating under HHP in both *in vivo* and *in vitro* conditions (Salmon, 1975a,b; Messier and Seguin, 1978; Bourns et al., 1988; Ishii et al., 2004; Arai et al., 2008; Nishiyama et al., 2010; Hayashi et al., 2016). A simulation study conducted by Dicken et al. demonstrated that the difference in Gibbs free energy between monomer and dimer of *Methanococcus jannaschii* FtsZ decreases with pressure, which could potentially explain the HHP-induced depolymerization of FtsZ (Dicken et al., 2015). Recent study suggests that depolymerization of microtubules induced by HHP is largely due to internal cavities and voids. Furthermore, compared to longitudinal interactions, the lateral interactions are more susceptible to HHP (Gao et al., 2018).

The functional Z-ring is a discontinuous structure composed of a bundle of FtsZ filaments formed through lateral filament interactions (Fu et al., 2010; Szwedziak et al., 2014). Earlier studies have demonstrated that lateral interactions enable the FtsZ protofilaments to associate and form the Z-ring structure at the division site. Disruption of the lateral interaction may lead to a failure of cell division (Monahan et al., 2009; Shin et al., 2013; Guan et al., 2018). The lateral interface of FtsZ is composed of residues in helices H3, H4 and H5 in the N-terminal GTPase domain, and the C-terminal variable region (Buske and Levin, 2012; Guan et al., 2018). In this study, we found that mutation of two residues in the helices H3 and H5 (L80A and E152A) of FtsZ_Sb_, which were close to the lateral interface, led to significant defect in HHP tolerance. In these two mutants, Z-ring-like fluorescence was observed in 5% of cells under atmospheric pressure, which was slightly less than the wild-type FtsZ_Sb_, but almost undetectable in cells incubated under 50 MPa. It is thus speculated that longer side chains of these two residues enhance the lateral interaction between FtsZ filaments under HHP condition, thereby reinforcing the stability of FtsZ filaments and the Z-ring.

In addition to the intrinsically driven lateral interactions, modulatory proteins such as ZapABCDE have been documented to promote the lateral interactions between FtsZ filaments and stabilize the Z-ring (Durand-Heredia et al., 2011, 2012; Galli and Gerdes, 2012; Marteyn et al., 2014; Schumacher et al., 2016, 2017). It is now understood that FtsZ interacts with ZapA through its C-terminal region and the two lysines in its GTPase domain are close to the binding site and are cross-linked with ZapA (K51 and K66 in FtsZ_Ec_, corresponding to K52 and K67 in FtsZ_Sb_). These two lysines are highly conserved across *Shewanella* species analyzed, regardless of whether they inhabit the shallow or deep water. However, our findings showed that residues between these two lysines may play a significant role in the adaptation to HHP. The septum localizations of mutant FtsZ S54G and T57S were notably impaired by HHP, as only half or one-third cells displaying Z-ring-like fluorescence under 50 MPa compared to cultures incubated at atmospheric pressure. One possible explanation for this is that these mutations disrupted the interaction between FtsZ and ZapA, thereby weakening the stability of FtsZ_Sb_. It should be noted that these residues are also in the vicinity of the GTP binding site, and further experimental evidence is required to elucidate their contribution in the HHP adaptation of FtsZ_Sb_.

Another possible factor influencing HHP tolerance could be the GTPase activity. Changes in GTPase activity can impact both the morphology and organization of FtsZ filaments, as well as the rate of treadmilling, which is essential for Z-ring assembly and cell division (Lu et al., 2001; Jaiswal et al., 2010; Arjes et al., 2015; Lyu et al., 2016; Yang et al., 2017). While there have been no previous reports on the effect of HHP on the GTPase activity of tubulin, numerous studies have demonstrated that the GTP binding and GTP hydrolysis of the guanine nucleotide binding proteins, such as those involved in cellular signaling pathways, are sensitive to elevated pressure (Siebenaller and Murray, 1994; Siebenaller, 2003). Given that several mutations in the GTPase domain of FtsZ_Sb_ led to decreased tolerance to HHP, we have attempted to directly measure the GTPase activity of different FtsZ proteins under elevated pressures. Unfortunately, accurately adding solutions of several microliters to the reaction system while maintaining pressure proved to be a significant challenge, leaving this hypothesis untested.

In this study, we noticed that after 24 h’ incubation under 50 MPa, cells expressing FtsZ_Sb_ were also elongated as the wild-type *E. coli* cells, even though the sharp Z-ring-like fluorescence could be observed (Supplementary Figures S5A2,A3,B2,B3). This observation suggested that a pressure tolerant FtsZ alone is not sufficient to restore cells division under high pressure condition. The complex process of cell division encompasses the positioning of FtsZ, protein recruitment and divisome assembly, constriction, hydrolysis and synthesis of peptidoglycan, with any disruption potentially leading to impaired cell division (Du et al., 2019). In addition, given that the majority of divisome components are anchored on inner membrane, it is not surprising that the physicochemical property of cellular membrane is also important for cell division (Kawamoto et al., 2009). It is well known that HHP decreases membrane fluidity which may impair the activity of membrane proteins and membrane associated bioprocesses (Welch and Bartlett, 1998; Abe, 2013; Tamby et al., 2023). Previous study showed that deep-sea piezophilic bacteria deficient of eicosapentaenoic acid are able to form Z-ring but fail in nucleoids partition and cell division under HHP condition. This suggested a disruption at the late stage of cell division (Kawamoto et al., 2011). The saturated fatty acids such as stearic acid and palmitic acid are the predominant fatty acids in *E.coli* cells (McGarrity and Armstrong, 1981). Meanwhile, monounsaturated acids account for 50–60% of total fatty acids in obligately piezophilic bacteria such as *Shewanella benthica* DB21MT-2 (Fang et al., 2004). The differences in fatty acids may also be a reason for the failure of division of *E.coli* cells under HHP. Therefore, even though FtsZ were correctly translocated to the septum and formed the Z-ring-like structure, the influence of HHP imposed on other protein components and membrane may impede division of *E. coli* cells under high pressures. Taken together, this study showed that FtsZ from the obligately piezophilic strain exhibits superior pressure tolerance and identified key residues for the adaptation of FtsZ to HHP. Yet, it is crucial to examine the impact of HHP on not only FtsZ but also other proteins involved in cell division, to fully comprehend this intricate process under extreme HHP environments.

## Data availability statement

The raw data supporting the conclusions of this article will be made available by the authors, without undue reservation.

## Author contributions

X-HC: Data curation, Formal analysis, Investigation, Methodology, Writing – review & editing. Y-CW: Data curation, Formal analysis, Investigation, Methodology, Writing – review & editing. X-GL: Methodology, Writing – review & editing, Supervision. X-QQ: Investigation, Project administration, Writing – review & editing. L-FW: Conceptualization, Funding acquisition, Supervision, Writing – review & editing. W-JZ: Conceptualization, Funding acquisition, Investigation, Supervision, Writing – original draft, Writing – review & editing.
