# Corrigendum: N-terminus GTPase domain of the cytoskeleton protein FtsZ plays a critical role in its adaptation to high hydrostatic pressure

**DOI:** 10.3389/fmicb.2025.1567029

**Published:** 2025-02-12

**Authors:** Xue-Hua Cui, Yu-Chen Wei, Xue-Gong Li, Xiao-Qing Qi, Long-Fei Wu, Wei-Jia Zhang

**Affiliations:** ^1^Laboratory of Deep-Sea Microbial Cell Biology, Institute of Deep-Sea Science and Engineering, Chinese Academy of Sciences, Sanya, China; ^2^College of Earth and Planetary Sciences, University of Chinese Academy of Sciences, Beijing, China; ^3^Institution of Deep-Sea Life Sciences, IDSSE-BGI, Sanya, China; ^4^Aix Marseille University, CNRS, LCB, Marseille, France

**Keywords:** obligate piezophile, cell division, cytoskeleton, FtsZ, high hydrostatic pressure, GTPase domain

In the published article, there was an error in the Funding statement. The funding statement for the National Natural Science Foundation of China was displayed as “NSFC4207612, 42176121 and 91751108”. The correct Funding statement appears below.

