# Supplementary material for: N-terminus GTPase domain of the cytoskeleton protein FtsZ plays a critical role in its adaptation to high hydrostatic pressure
Source: Front Microbiol. 2024 Aug 16;15:1441398. doi: 10.3389/fmicb.2024.1441398 (PMC11362102; doi:10.3389/fmicb.2024.1441398)
Supplement: Supplementary file 1 [file Table_1.DOCX]

Supplementary Material

N-terminus GTPase domain of the cytoskeleton protein FtsZ plays a critical role in its adaptation to high hydrostatic pressure

Xue-Hua Cui^1,2^, Yu-Chen Wei^1^, Xue-Gong Li^1,3^, Xiao-Qing Qi^1,3†^, Long-Fei Wu^1,3,4*^ and Wei-Jia Zhang^1,3*^

^1^Laboratory of Deep-Sea Microbial Cell Biology, Institute of Deep-sea Science and Engineering, Chinese Academy of Sciences, Sanya, Hainan, China

^2^University of Chinese Academy of Sciences, Beijing, China

^3^Institution of Deep-sea Life Sciences, IDSSE-BGI, Sanya, Hainan, China

^4^Aix Marseille University, CNRS, LCB, Marseille, France

^†^Current address: Hainan Research Academy of Environmental Sciences, Sanya, Hainan, China

*** Correspondence:**Dr. Wei-Jia Zhang
[wzhang@idsse.ac.cn](mailto:wzhang@idsse.ac.cn)

# Supplementary Figures


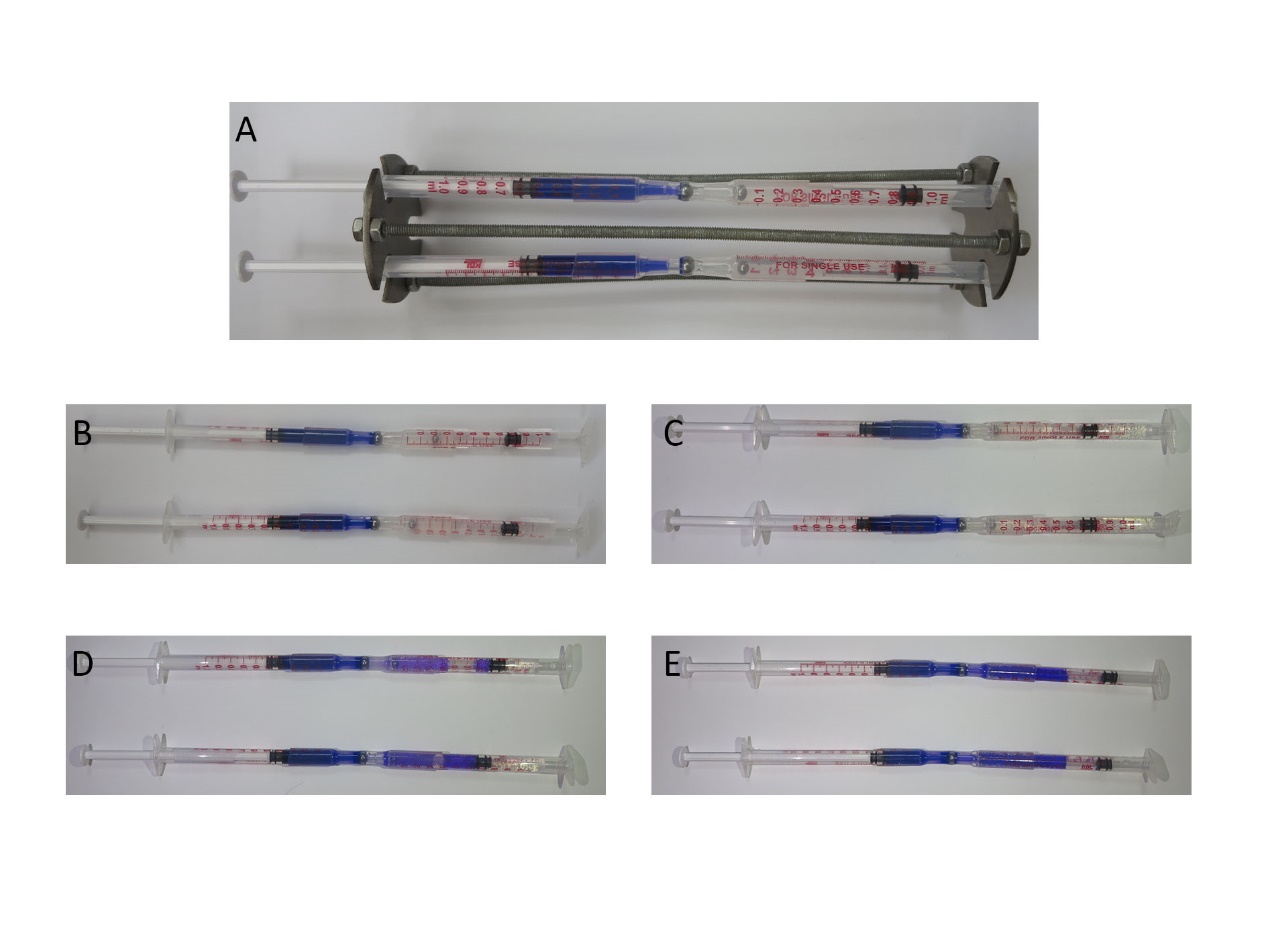


**Supplementary Figure 1.** The HHP *in situ* fixation apparatus and the injection of fixative

Panel A shows photo of core unit with two sets of syringes on the syringe rack. Panel B to E show the effect of injection of fixative from syringe A into syringe B by injecting 30 mL (B), 40 mL (C), 50 mL (D) and 60 mL (E) of water from the left inlet of HP vessel under 50 MPa, respectively.


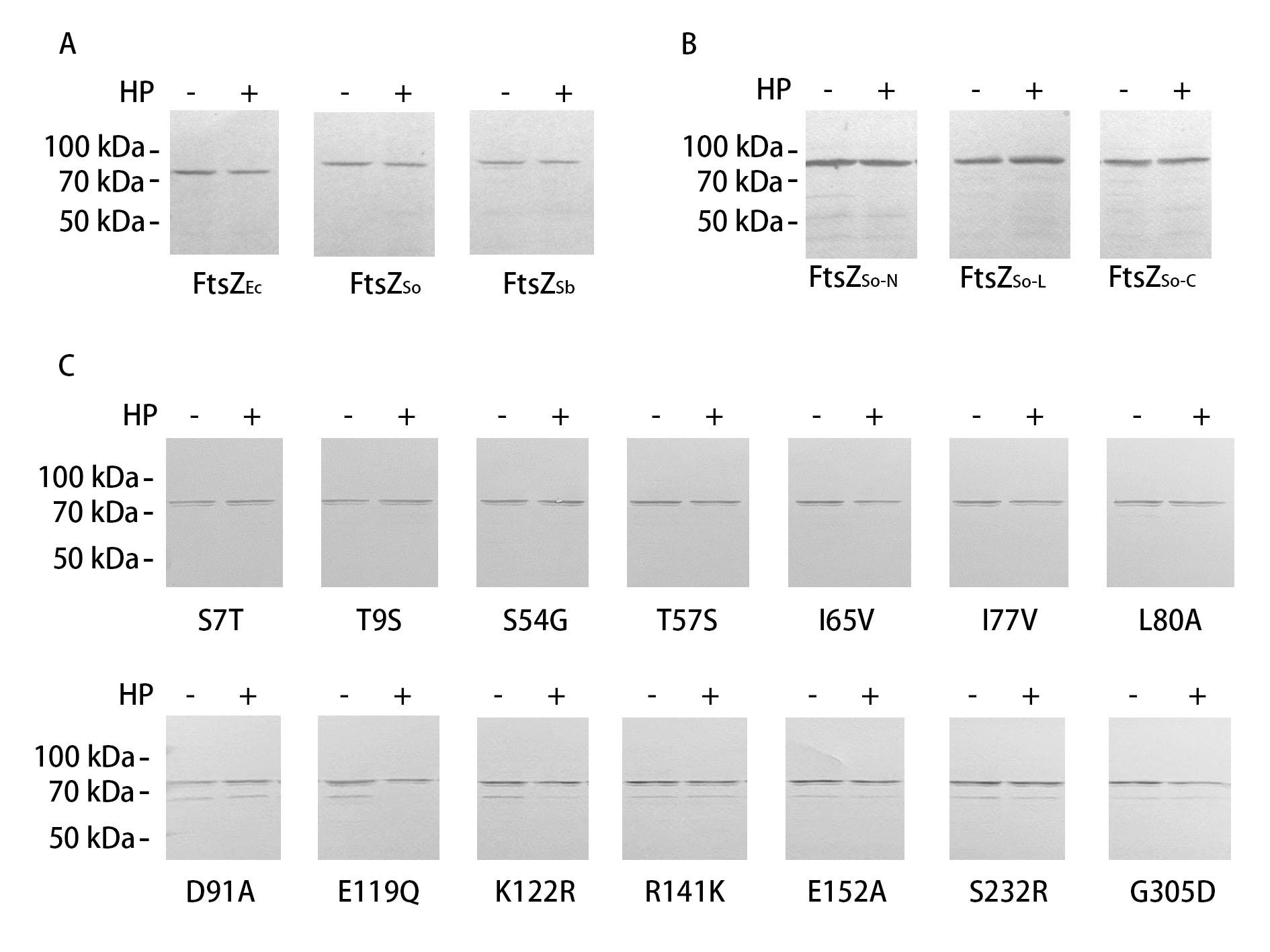


**Supplementary Figure 2.** Expression of FtsZ-GFP fusion proteins under different pressures

Panel A shows expression of FtsZ_Ec_, FtsZ_So_ and FtsZ_Sb_ under atm (0.1 MPa) and HP (50 MPa) conditions. Panel B shows expression of chimeric FtsZ proteins. Panel C shows expression of mutated FtsZ_Sb_ proteins.


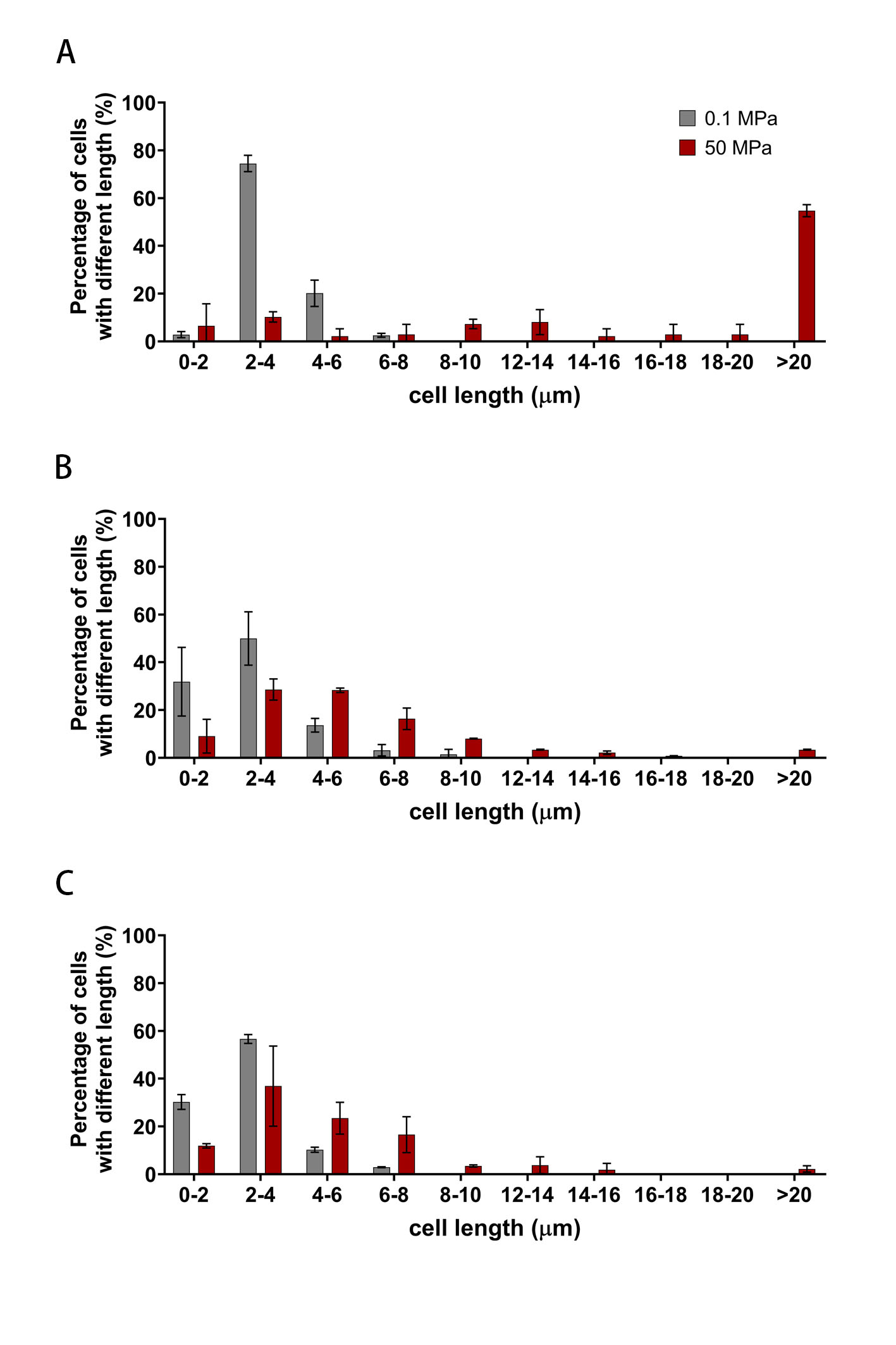


**Supplementary Figure 3.** Effect of HHP on cell length of *E. coli* cells expressing different FtsZ proteins

Panel A, B and C show distribution of cellular length of *E. coli* expressing FtsZ_Ec_, FtsZ_So_ and FtsZ_Sb_, respectively. The grey bars represent cultures incubated for 5 hrs under atmospheric pressure and the red bars represent cultures incubated for 5 hours under 50 MPa. The data were collected from three independent assays, in each assay over 300 cells were measured.


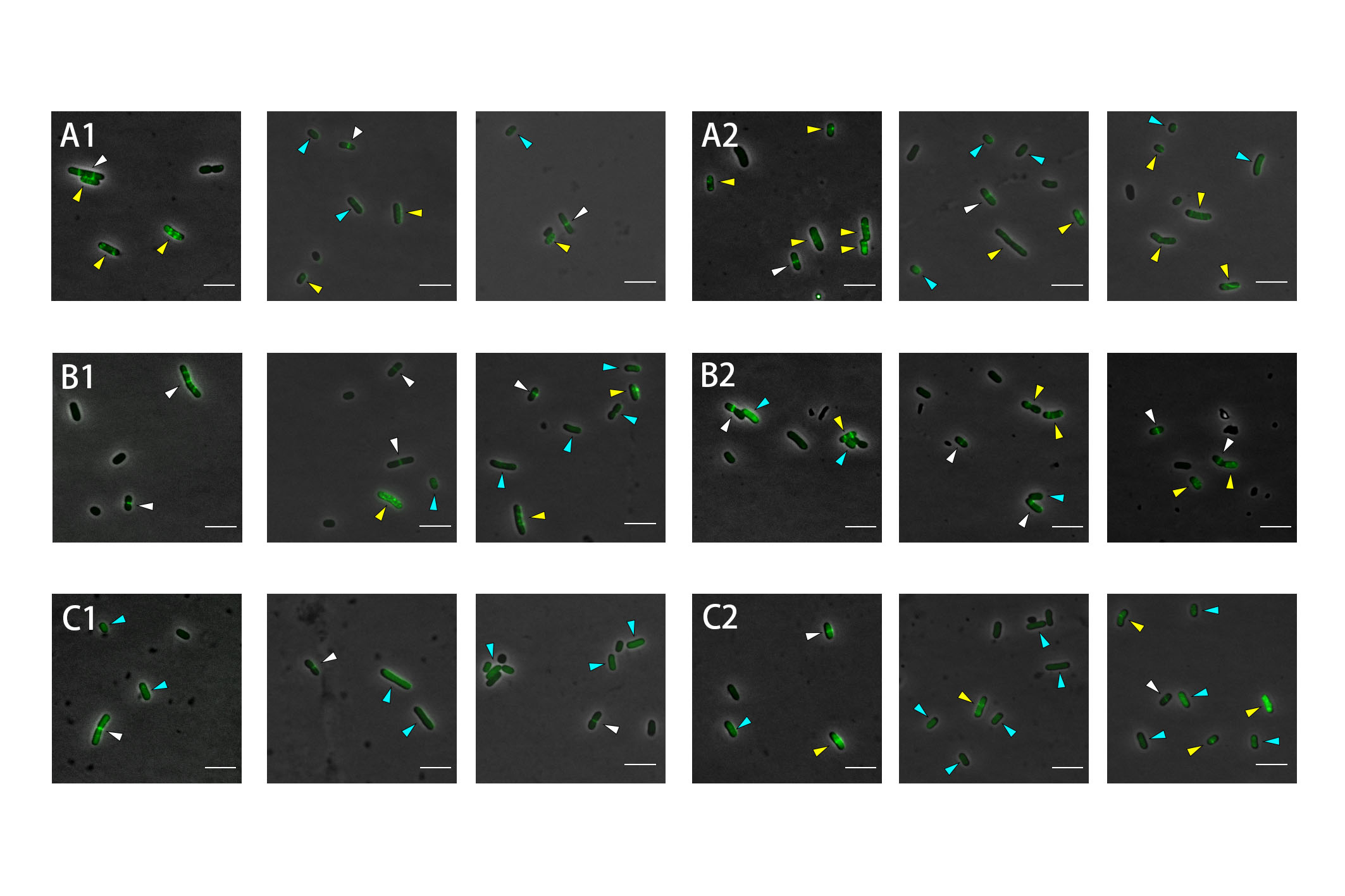


**Supplementary Figure 4.** Effect of HHP on subcellular localization of FtsZ_Ec_, FtsZ_So_ and FtsZ_Sb_

Panel A1 and A2 show fluorescence of FtsZ_So-N_-GFP under 0.1 MPa (A1) and 50 MPa (A2); Panel B1 and B2 show fluorescence of FtsZ_So-L_-GFP under 0.1 MPa (B1) and 50 MPa (B2); Panel C1 and C2 show fluorescence of FtsZ_So-C_-GFP under 0.1 MPa (C1) and 50 MPa (C2). All scale bars indicate 5 μm. The white arrows indicate cells with Z-ring-like fluorescence, blue arrows indicate cells with homogenous fluorescence and yellow arrows indicate cells with ectopic fluorescence.


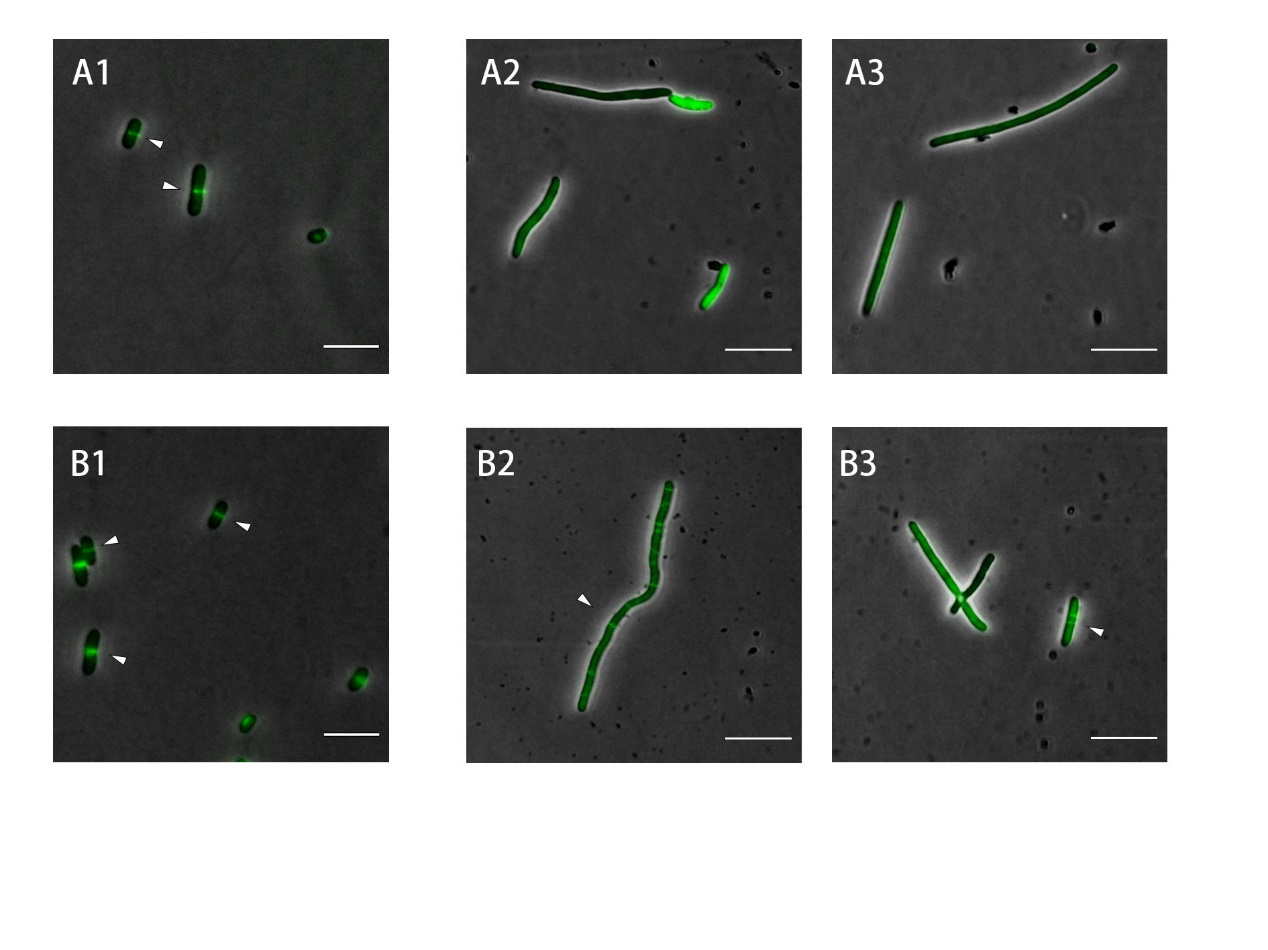


**Supplementary Figure 5.** Cellular morphology and FtsZ localization after long-term incubation

Panel A1 to A3 show *E. coli* cells expressing FtsZ_So_-GFP after 24 hours’ incubation at 0.1 MPa (A1) and 50 MPa (A2 and A3). Panel B1 to B3 show *E. coli* cells expressing FtsZ_Sb_-GFP after 24 hours’ incubation at 0.1 MPa (B1) and 50 MPa (B2 and B3). The scale bar indicates 5μm.
